# Students' perceptions about the transition to the clinical phase of a medical curriculum with preclinical patient contacts; a focus group study

**DOI:** 10.1186/1472-6920-10-28

**Published:** 2010-04-05

**Authors:** Merijn B Godefrooij, Agnes D Diemers, Albert JJA Scherpbier

**Affiliations:** 1 School for Public Health and Primary Care (CAPHRI), Department of General Practice, Faculty of Health, Medicine and Life Sciences, Maastricht University, Maastricht, the Netherlands; 2 Skills lab, Faculty of Health, Medicine and Life Sciences, Maastricht University, Maastricht, the Netherlands; 3 Institute for Education, Faculty of Health, Medicine and Life Sciences, Maastricht University, Maastricht, the Netherlands

## Abstract

**Background:**

Studies have shown that medical students experience the transition between preclinical and clinical training as a stressful period. They are generally frustrated by their inability to apply their knowledge to solve clinical problems in practice. Preclinical patient contacts may offer a solution to this 'shock of practice.' We studied how students who have had preclinical patient contacts perceive the transition from preclinical to clinical training and, more specifically, how they value these early patient contacts as preparation for learning in clinical practice.

**Methods:**

A purposive sample of 21 students participated in three focus groups which met twice during their first weeks of clinical clerkships. The interviews were recorded and transcribed literally. Qualitative content analysis of the transcriptions was performed.

**Results:**

According to the students, working in clinical practice was enjoyable, motivated them to study and helped them to develop non-analytical reasoning skills. The students experienced stress due to increased working hours and work load, uncertainty as to what was expected of them and self-perceived lack of knowledge. They did not experience a major gap between the preclinical and clinical phase and felt well prepared for the clerkships. The preclinical patient contacts were considered to be instrumental in this.

**Conclusions:**

Early patient contacts seem to ameliorate the shock of practice and prepare students for clinical work. The problems mentioned by the students in this study are mainly related to the socialisation process. The results of this study have to be validated by quantitative research.

## Background

The transition between the theoretical and the clinical phase of undergraduate medical education has often been characterised as the most stressful period of undergraduate medical education [1-3]. The first clinical year has been described as a period where medical students go through intense emotional experiences [4] and students have described entering the clinical arena as though they were being "thrown in at the deep end" [5]. Boshuizen [6] highlighted that the "shock of practice", a crisis experienced by many medical students on first entering the clinical workplace, is marked by a temporary decrease in their ability to properly use biomedical knowledge in clinical reasoning.

The ability to use theoretical knowledge to solve clinical problems is claimed to be enhanced by Problem-Based Learning (PBL) [7]. It has been suggested that the transition from theory to practice is less problematic for students in a PBL curriculum. However, both Van de Wiel *et al*. [8] and Prince *et al*. [9] discovered that, despite the use of PBL, students from a PBL-based medical school experienced difficulties that were similar to those reported by students from more traditional curricula. Early patient contacts are advocated as a way to improve students' preparedness for clerkships and hence overcome the "shock of practice" [9-12]. As yet there is not much evidence of the effects of these contacts.

An opportunity to examine such evidence was presented by the introduction of preclinical patient contacts in the Maastricht PBL curriculum. We explored the following research question: how do students who have had preclinical patient contacts perceive the transition from preclinical to clinical training and, more specifically, how do they value these early patient contacts as preparation for learning in clinical practice?

## Methods

### Research method

We explored students' perceptions of the transition from the preclinical to the clinical phase through focus group interviews. Focus groups are widely used in exploratory and qualitative education research [13] to gain insight into participants' perceptions, opinions and the processes underlying them. Focus groups can elucidate both *what *participants think and *why *participants think as they do [14]. Participants are encouraged to react to one another's views and generate new ideas from different perspectives, something that is not possible in one-on-one interviews [14].

### Context of the study

Since the foundation of the Maastricht Faculty of Medicine in 1974, PBL has been the predominant educational approach. In 2001, a curriculum renovation was implemented to further the integration of theory and practice by a gradual increase of practice-based activities and a concomitant decrease in theory-based activities from year 1-6. Additionally, early contacts with real patients were introduced in Year 3, while time for basic sciences and reflection on clinical experiences was included in the clerkships in Years 4 and 5.

Currently, the first two years of the curriculum consist mainly of six- to ten-week thematic units during which students work in tutorial groups with paper patients as the starting point for their learning. During those first two years students only occasionally see real patients during lectures, or on videos during tutorial group meetings. In Year 3, the paper patients are replaced by weekly encounters with real patients. Students prepare for these by discussing relevant vignettes, describing the problem of the patient, in small groups one or two days before seeing a patient in the outpatient teaching clinic of Maastricht University Hospital. Student couples are observed by the patient's attending physician while taking a history and performing a physical examination. After the encounter the physician discusses the patient contact with the students, provides feedback on their performance and guides them in deriving learning issues. The attending physician remains responsible for the treatment and management of the patients' disease. The derived learning issues direct students' self-study activities, which are supported by other educational activities, such as lectures, lab work and skills training, including communication skills training with simulated patients. One week after the patient contact, the students present their patient and discuss the results of their self-study in the next group session.

An extensive description of Year 3 of the Maastricht curriculum can be found elsewhere [15].

Although the order differs per student, clerkship rotations in Year 4 are in dermatology, ear-nose-throat (ENT), ophthalmology, internal medicine and surgery plus an elective clerkship, followed in Year 5 by paediatrics, obstetrics and gynaecology, community medicine, neurology, psychiatry and general practice. During the rotations in dermatology, ENT and ophthalmology days in the hospital alternate with days devoted to teaching and reflection in order to ensure that the main subjects of these disciplines are taught alongside workplace experiences. Most of the other rotations are preceded by an introductory week where prior knowledge and skills are activated and conclude with a final week for reflection, reporting and assessment. Clerkships are offered in Maastricht University Hospital and affiliated teaching hospitals. The sixth and final year of undergraduate training is divided into an eighteen-week research project and eighteen weeks of participation in patient care as a junior doctor.

During the above described curriculum, students follow a longitudinal skills training programme in the skills laboratory from year 1-5. During the programme they practise skills (including communication skills) on models, manikins, each other and simulated patients.

### Participants

At the start of the academic year 2006-2007 all 294 fourth-year students were invited by email to voluntarily participate in focus groups. A reminder was sent by email after one week. Of the 32 students who responded, seven were excluded because they did not meet the condition of having completed at least one full week of their first rotation at the time of the first session. The remaining 25 students were assigned to one of three focus groups according to availability and rotation. In order to achieve maximal variation in disciplines and teaching hospitals, we set the maximum number of students in the same rotation per group at three. This resulted in the exclusion of two students. Because two other students, for different reasons, withdrew their participation, the final three focus groups consisted of six, eight and seven students, respectively. One student of group 2 was unable to attend the second focus group session of group 2, and participated in the second session of group 3 instead. One student of group 3 was unable to attend the first session.

The participants were on rotations in all the disciplines of Year 4 (Table 1). Of the participants 67% (n = 14) were female, which reflects the gender distribution of this Year 4 cohort. Clerkship experience of the students ranged from one to three weeks at the time of the first focus group and by the second session some students had finished their first rotation and moved to a different discipline. The students received a financial incentive of 30 euros for participation and for critically commenting on the summary of the first interview and the final report. They were assured that the results would be reported anonymously.

**Table 1:** An overview of the participants in each group at the time of the 1^st ^and 2^nd ^interview

*Clerkship*	*Group 1*		*Group 2*		*Group 3*	
	***1*^*st*^**	***2*^*nd*^**	***1*^*st*^**	***2*^*nd*^**	***1*^*st*^**	***2*^*nd*^**

Dermatology	1	0	2	1	1	1

ENT	1	3	1	2	1	1

Ophthalmology	3	1	3	1	2	1

Internal medicine	1	1	1	1	2	3

Surgery	0	0	1	2	0	2

No current clerkship	0	1	0	0	0	0

### Procedure

The focus groups met twice, with a one-month interval, between September and November 2006. After the second session saturation was deemed to have been reached, so no third session was organised. The meetings were scheduled for 1.5 hours after working hours to avoid interference with students' clinical work. The discussions were guided and stimulated by the first moderator (AS), who is familiar with the curriculum and a highly experienced moderator of focus groups. The assistant moderator (MG) took notes and made sure the sessions were audio taped. Occasionally, he asked the participants to clarify statements.

### Instruments

For the first session a set of open-ended questions was prepared relating to the following topics:

1. Students' experiences during their first clerkship week.

2. Perceived differences between preclinical and clinical training.

3. The role of the preclinical patient encounters in the students' overall preparation for clinical training.

4. Recommendations to further ease the transition from preclinical to clinical training.

During the second session the findings of the first session were verified and clarified and the participants were asked to comment on a written report of the first session, that was send to them in advance of the second session. Additionally, the role of Year 3 as preparation on the clinical phase was elaborated upon.

### Data collection and analysis

All focus group sessions were audio taped and transcribed literally by MG. Using the software program ATLAS.ti [16], coding of text fragments based on content was done through multiple coding by three researchers (MG, AD and AS) independently [17]. They then compared the coded text fragments and adjusted their coding until consensus was reached. Subsequently, the codes were reorganised and grouped, main themes and sub-themes were identified and illustrative quotations selected. The final report was sent to all participating students for approval. All the students responded and approved the summary of the first interview and the final report, without any comments or modifications.

### Ethical approval

At the time of the study, educational research studies reporting students' opinions did not require approval from the ethics committee in the Netherlands. However, relevant ethical issues were carefully considered by the Chair of the Department of Educational Development and Research at Maastricht University.

## Results

Guided by the open-ended questions, the three groups discussed almost identical topics. We present the main themes and the sub-themes (Table 2) with illustrative quotations. Differences of opinion in the groups are reported.

**Table 2:** Main themes and corresponding sub-themes discussed during interviews

Experiences	Experiences during first week Transition from Year 3 into the clerkships Role as a clerk Emotions Amount of structure offered during clerkship Workload and working hours Contact with other health professionals
**Knowledge and skills**	Level of knowledge Basic science knowledge Clerkship as a place for learning Preparation for clinical skills Integration of clinical skills Introductory week

**Clinical reasoning**	Clinical knowledge and reasoning skills Analytical reasoning and processes of recognition Diversity of patients Recognising pathology

**Learning**	Motivation Learning during clerkships Clinically oriented studying Balance between theory and practice

**Preclinical patient encounters**	Motivation Self-confidence Sense of responsibility Clinical reasoning skills Bridging a gap

### Experiences

The students' experiences during the first week of clinical training varied but the prevailing view was that they thoroughly enjoyed the experience.

"Working as a clerk is better than expected" (FK1)

"I did not look forward to the clerkships, but now that I'm in it: it really is one of the best experiences I have ever had." (JR3)

One of the most dramatic differences with preclinical training was the huge increase in working hours, although students in each group also said this was to be expected on moving from preclinical to clinical training.

"Yes, all that busyness. All at once your days are full. Now, during the winter period, I feel like some kind of caveman: you go to work in the dark and you come home in the dark. That really is a change for me." (FK1)

"If you do any other study there would also come a point where you'd have to start gaining work experience (...) and you start working, so it's just all in the game." (KN2)

Not only the hours but also the intensity of work increased for most of the students. The work pace in the hospital was high, which meant less time for individual patients, studying and homework assignments. Some of the students whose first rotation was in a rotation with alternate clinical and reflection days (dermatology, ophthalmology and ENT) did not yet experience a great difference in workload, but they anticipated this would happen when they moved to other rotations, like internal medicine or surgery.

A challenge reported by most students was getting to grips with their role and position in the clinical department: how were they supposed to behave and act? Occasionally, it was difficult for them to understand what staff expected from them. In most hospital settings there seemed to be a set of unwritten rules for clerks.

"What I find difficult to sense is, you know, when it is okay to speak up and when you had better keep quiet." (MB1)

"I think what we mean here is this: you are put on a ward, which is challenging, but you don't know the rules. You don't know which person is nice and which person is not, and sometimes even how you should dress (...)." (CS2)

Students indicated that it would be easier for them to find their place in a department when clerkships were more structured. They were eager to understand where they were expected to be and at what time. However, this uncertainty was not experienced as negative by all the students. Some students appreciated their freedom to determine their own schedule.

"No, I do not have a problem with being thrown in at the deep end, but structure... so that you know what's being expected of you, that I would appreciate." (RS2)

"(...) Apparently there are these meetings going on everywhere in the hospital, but where do I have to run to, and at what time?" (MK2)

"When there are moments when it's very quiet I actively start looking for something to do. (...) Now I have the chance to see those patients, so I just go in search of them." (CB2)

Students pointed to the emotional impact of having to deal with many patients with serious illnesses as well as their increased sense of responsibility for patients. They admitted they had to learn to cope with these - at times very intense - emotions.

"What I find a bit heavy of the clerkships is how many impressions you get. You see so many sick people in such a short period of time. And that does affect you at first, so that sometimes you think: wow, these are more people than I expected, it's all a bit worse than I expected, and that makes you think." (MK2)

### Knowledge and skills

Upon entering the clinical environment, students are confronted with their own very limited knowledge compared to that of clinicians:

"You enter this environment with these medical doctors and professors, and you feel they know everything. And quite possibly you actually know quite a lot, but because of this environment with all these doctors (...), you can get that feeling." (MG2)

"I haven't even finished my second week as a clerk yet. I'm currently going through the absolute depression of ignorance." (MK2)

Many students experienced deficiencies in basic science knowledge and in their ability to apply it. Deficiencies in anatomy, pharmacology, physiology, endocrinology and the interpretation of lab results were mentioned and confirmed by the majority of the students.

"Anatomy is the very worst" (FK1)

"It is important that you have some kind of basis, and we don't have that." (CS2)

"There's a lot you don't know yet. I find it very frustrating when I am asked a question and again and again I'm not able to give the correct answer." (KN3)

These reactions were qualified, however. For instance, students acknowledged that clerkships are for learning, implying that it is normal for them not to know everything yet. Students also observed that although the use of pharmaceutical brand names was difficult, they did understand most of the mechanisms of the drugs prescribed by doctors. Furthermore, some anatomical knowledge proved to be easier learnt from real patients than from textbooks:

"I'm a clerk in order to learn, if I knew everything already then I wouldn't be here" (KN2)

"On my first day I didn't know exactly what veins and arteries looked like. But within one day that knowledge was drilled into me, and then I knew exactly what they looked like. You only need to see two blood vessel operations in order to know exactly how all these veins run through the human body. And then you also understand their relation with other structures, which I find difficult to learn from textbooks." (JR3)

The confrontation with deficiencies in knowledge was a powerful drive for the students to study and they thought deficiencies could be interpreted as learning issues to guide their studies.

In general students felt well prepared with regard to clinical skills, communication skills in particular. However, it was confusing for the students when different doctors had different notions of the correct way to perform a physical examination:

"But you can never do it right, because each doctor has his own method and says: no, you have to do it this way. And next time, when you do it like that, another doctor will tell you: no, you should do it this way." (KN3)

Another challenge in respect of clinical skills was to integrate skills students had learned as separate entities into a smooth physical examination. However, this was easily remedied thanks to an extra training session during the introductory week of some rotations.

"The only disadvantage is that we learn everything in packages, you know: examination of the heart, examination of the lungs... And in internal medicine you need to do all these examinations in one and the same patient. And when you first do a full cardiologic examination and then a full pulmonary examination, your patient has to turn over six or seven times." (MG1)

"I think we've had enough practice [with regard to clinical skills]. I thought it was really good that a full physical examination was demonstrated during the introductory week of internal medicine." (JB3)

Finally, the students valued the introductory weeks of most rotations, which helped them refresh and integrate their prior knowledge and skills and fill the most important gaps in their knowledge, thereby improving their preparedness for the rotation. Nevertheless the students also noted a need for improvement in the educational quality of the introductory week in some rotations.

"You are really stimulated to refresh your knowledge, for instance in cardiology and other subjects... That is really good." (CB2)

"I don't think lectures are the ideal way to teach students for eight hours a day." (JG3)

### Clinical reasoning

The students perceived a shift of emphasis from theoretical knowledge in the preclinical years to clinically oriented knowledge and reasoning in the hospital. The emphasis moved from understanding underlying mechanisms of disease to recognising clinical signs and symptoms and making treatment decisions.

"Last year treatment was not our main goal, you know, but now the emphasis is more on management strategies. That's why it's more important now to be up-to-date. Before you would say: you can either operate or you can laser it, but now the question is: how exactly do you operate or laser it?" (FM3)

"I think, from now on, all we will be doing during our clerkships is work on those differential diagnoses." (FK1)

The students said that pattern recognition became increasingly important, sometimes at the expense of analytical reasoning. They also said that most doctors did not encourage them to fully understand the underlying mechanisms of a disease, and that often they were not given enough time to fully exploit their analytical reasoning skills.

"Because you are so much busier during the clerkships, you are already pleased when you recognise something. And I think that will become more and more important, because in the outpatient clinic doctors never ask you to explain symptoms. If you recognise symptoms, they are usually quite pleased with you already, and often they themselves don't understand the exact underlying mechanisms." (JP1)

The number of patient encounters was much higher than during the preclinical years. Students appreciated the diversity of patients they saw, because it enabled them to compare symptoms and diseases and expand their reference base for recognition processes.

"And now you just see more patients in one day, and you can compare patients: one patient deals with his problem like this, the other patient like that. And: this one has this much inconvenience, and that one that much. Which symptoms coincide, and which differ enormously?" (JP1)

"But I also find it easier, because you have seen patients and you can make connections, and then you recognise things faster - that is a great advantage." (SP1)

The students said that patient encounters provided a frame of reference for identifying physiological and pathological processes, although it was sometimes difficult for them to recognise pathology.

"It's very useful that you get these frames of reference about what's normal and what's not, especially with auscultation. Sometimes you think you hear something abnormal and then you think: hey, I've found something! Then you go to the doctor and then: no, that's nothing. But then you know the next time, that it's nothing, so that's useful." (JR3)

### Learning

There was general agreement that motivation to study increased during clerkships. Studying was more fun. The main motivators were patients and doctors. Students enjoyed being able to apply their knowledge in practice.

"It is so cool and you learn so much, and there's so much that you can do yourself." (MB1)

"When those doctors ask you questions then you don't want to make a fool of yourself. So yes, that motivated me to really study some subject-matter." (LW1)

"Your learning improves: the fact that you have a patient contact makes you want to know things better." (JW3)

"It's also more fun to notice that you can apply the things you learn." (FM3)

However, it was also remarked that in-depth studying was discouraged if clinicians had low expectations of students:

"It is true that your attitude becomes more lax, [if these people are so easily satisfied], so you think: I don't really have to study that chapter because they won't pick me up on it anyway tomorrow, so I will go and have another beer now." (FM3)

The students experienced an increase in the speed and ease of learning. They saw more patients and more diseases than before and this repetition made it easier for them to memorise knowledge about various diseases.

"It sticks in your mind more easily when you are working with a patient" (KN3)

"You see 10 times as many patients, so it just goes much faster" (MB1)

According to the students, learning issues were less broad and more specific. The increased patient load meant less time to study for each case. Some students perceived this more specific way of studying as detrimental to in-depth studying.

"As far as knowledge goes, I think I learn less from [each patient now]. Because at the end of the day I have a list of at least 10 things that I could look up but very often I don't even get to that anymore" (MG2)

"And much more specific (...) I often look for one specific thing (...) Specifically for the use of that particular drug when I run into that. I don't study in the way I used to: an entire disease with its epidemiology and then symptoms, diagnosis, treatment, no longer in that order." (MG1)

The students agreed that learning - like their knowledge - was more clinically oriented now. They hardly ever opened a physiology textbook and the importance of keeping up with recent literature and publications had increased.

"Yes, a lot more articles. When I read those books, I come in and think: oh, that's a good thing to mention. But then they say: that is so out-dated." (JR3)

Most students were happy with the combination of theory and practice and preferred practice, although they agreed that it was also important to have sufficient time for studying and reflection.

"The combination of theory and seeing patients, to be able to apply it all, that's a lot of fun." (MK2)

### Early patient encounters

Students generally felt better prepared for clinical training as a result of the patient encounters in Year 3, which had given them more self-confidence in dealing with patients and helped them to develop interview and physical examination skills in a safe environment.

"Because you have already done it a couple of times (...) then [at the beginning of the clerkships] you feel: I'm ready for it. I also felt like I wanted to see patients because it's fun and because I'm not nervous about meeting them at all. And the more you do it, the better it goes and so you notice your confidence increases and your uncertainty disappears." (JW3)

"The thing that's really good about year 3 is those [early patient contacts]. Because now we have entered clinical practice it's so different from last year. Then there was so much calmness, you were really given the time and space you needed with your patient, and you were really well guided." (CS2)

The preclinical patient encounters had also been a strong motivation for students to study and taken them closer to their final goal: working as a medical professional. Students remarked that being more actively involved in their learning had made the preclinical patient contacts very enjoyable.

"Finally it's beginning to get professional. Finally it's moving toward being a doctor." (JP1)

"It really stimulated me to start studying, when you would see a patient and think: whoops..." (CS2)

Some students said that the preclinical patient contacts had triggered the development of a sense of responsibility, which was growing stronger during clerkships.

"Yes, and I think that because of that you already have a sense of responsibility. That in year 3 you really (...) are confronted with a patient." (HL2)

"In year 3, on our first time in the outpatient clinic we had prepared something but not everything. And then you feel: this patient has come here especially for you and you don't even know what to ask. And then you realise that you have a certain obligation towards patients, because they are so kind as to help you, you have to make sure you are well prepared." (FM3)

The benefit of the preclinical patient encounters that was mentioned most frequently was the development of clinical reasoning skills. The students said they had been challenged to build knowledge structures and experiment with differential diagnosis in a safe environment.

"What I like about year 3 is, and you notice that more and more now: it helps you to create a certain structure in your way of thinking. In year 3 we first made differential diagnoses and that is one of the most important things in medicine." (JB3)

"Year 3 was one of the best, or so to say, and it really helped me to learn to form all these connections into a comprehensive structure in my mind." (JP1)

Finally, there was general agreement that the preclinical patient encounters had eased the transition to clinical training and bridged the gap between the more theoretical orientation of preclinical training and the clinical orientation of the clerkships.

"But what is difficult sometimes is that you have learned to first study a disease and then its symptoms, and now you have to reason the other way around and sometimes that's difficult." (KN3) "But I think the good thing about year 3 is that it taught us how to deal with that." (JB3)

"Now we make the same kind of patient reports [as we did in year 3], we still have the same kind of learning issues to study, and we still use patients to link our knowledge to." (MK2)

"Year 3 really is a kind of pre-clerkship. Really a year in which you are prepared for clinical training." (MG2)

## Discussion

In this study we asked students whose preclinical curriculum had included encounters with real patients to talk about how they experienced the transition from the preclinical to the clinical phase of medical training. The general feeling among students was that they enjoyed their clinical experiences, especially the ability to put their knowledge into practice. Students felt well prepared for clinical practice and did not feel daunted by a large gap between preclinical and clinical training. Negative experiences were related to professional socialisation processes, the increased workload, and perceived knowledge deficiencies. Although these feelings differed somewhat amongst the students, they were largely deemed to be a normal aspect of entering a new work environment.

The most striking result of this study is that the "shock of practice" described by Boshuizen [6] and Prince *et al*. [12] in earlier studies was not reflected in the perceptions of the students in this study. Although the students reported a shift of emphasis from theoretical knowledge to clinical knowledge, they also said this transition was a gradual and natural process and the encounters with real patients in the previous year had enabled them take the first steps in a protected environment. In line with other research [12,18], the students thought the early patient contacts had increased their self-confidence, motivated them to study, helped them develop clinical reasoning skills, and inculcated a sense of responsibility. The last pre-clinical year was literally described as a bridge between preclinical and clinical training. It thus seems that early patient encounters are effective in counteracting at least part of the negative effects of the shock of practice.

Concerning the development of clinical reasoning skills, students noted that non-analytical reasoning became more prominent during clinical training and was facilitated by the rapid expansion of their 'reference database' as a result of the increasing numbers of patients they saw. Eva [19,20] has argued that non-analytical processes of reasoning are in no way inferior to more analytical forms of reasoning, but he and Ark also contended that the combined use of both reasoning strategies promotes diagnostic accuracy [20,21]. This suggests that both types of reasoning should receive attention during medical education. From this perspective it seems somewhat worrying that the students in our study felt they were not sufficiently guided in developing skills for analytical reasoning. This is the more reason for concern in light of Eva's [19] and Van de Wiel *et al*.'s [8] finding that students do not automatically engage in analytical reasoning of their own accord, but have to be explicitly alerted to links between theory and practice. This is supported by the students of our study, who said that feedback and questions from clinicians can be an important motivator and guide in the development of analytical reasoning and regretted that there often was not enough time for this.

It was also observed in earlier studies that students experience deficiencies in basic and clinical science knowledge when they are confronted with diseases and diagnostic methods that are new to them and with vastly more experienced and knowledgeable clinicians [9]. Prince *et al*. [22] also showed that these perceived deficiencies should not necessarily be interpreted as shortcomings of the curriculum. Although students from the Maastricht PBL curriculum felt they were vastly lacking in anatomical knowledge, Prince *et al.* demonstrated that their knowledge was in no way inferior to that of students from other medical schools. Additionally, Van Hell *et al*. [23] found that the levels of pre-clinical knowledge and skills did not influence students' performance during the transition period. Although experienced by the students, these deficiencies do not need to be a cause for direct concern. As some of the students acknowledged, clerkships are learning experiences and it is normal that their knowledge is imperfect. Nevertheless, it is important not to dismiss students' feelings in this respect.

One of the limitations of this study is that, although the amount of patient contacts during Years 1-3 is the same for all students, the amount of patient contacts during the start of Year 4 may differ and thus may influence students' experiences with the transition phase.

Another limitation is that the students might have known the main moderator as the head of the Institute for Medical Education. This may have withheld students from fully sharing their ideas and opinions. On the other hand, it may as well have encouraged them to express their feelings and ideas in order to suggest improvements to be made about the curriculum.

Furthermore, it has to be taken into account that the students who reacted on the invitation to participate in this study were probably the most enthusiastic ones.

Finally, the results of this study are based upon the opinions of a small sample of 21 students. Even though saturation was reached in all three groups and the groups discussed similar themes, it cannot be ruled out that not all relevant themes were appropriately covered. Nevertheless, the purposive sample ensured the inclusion of a wide variety of experiences. How representative the positive results are for the entire student population will have to be investigated by larger, quantitative studies, for example through a questionnaire survey amongst fourth-year students.

## Conclusions

The main conclusions from this study are:

- Early patient contacts seem to alleviate the perceived "shock of practice" during the transition from the pre-clinical into the clinical years of medical education and prepare students for their work as a clerk.

- The negative experiences regarding the transition period that were expressed by the students are mainly related to professional socialisation processes.

- Non-analytical reasoning processes become more important during the clinical years of medical education. Students often did not feel sufficiently challenged by clinicians to fully exploit their analytical reasoning skills.

The results of this study have to be validated by quantitative research.

## Competing interests

The authors declare that they have no competing interests.

## Authors' contributions

MG conceived and designed the study, participated in the data collection and analyses and drafted the original manuscript. AD made important contributions to the study design, participated in the data analyses and critically revised the original manuscript. AS made important contributions to the study design, participated in the data collection and analyses, and gave critical comments to the manuscript. All authors read and approved the final manuscript.

## Authors' information

MG (MD) was a sixth year medical student in the previous Maastricht curriculum, at the Faculty of Health, Medicine and Life Sciences at Maastricht University at the time of the study.

AD (MD) works at the skills lab of the Faculty of Health, Life Sciences and Medicine at Maastricht University. She holds a Master of Health Professions Education.

AS (MD, PhD) is professor of Medical Education and Scientific Director of the institute for Medical Education, Faculty of Health, Medicine and Life Sciences at Maastricht University.

## Pre-publication history

The pre-publication history for this paper can be accessed here:

http://www.biomedcentral.com/1472-6920/10/28/prepub
